# Effects of fire and fire intensity on the germination and establishment of *Acacia karroo*, *Acacia nilotica*, *Acacia luederitzii *and *Dichrostachys cinerea *in the field

**DOI:** 10.1186/1472-6785-4-3

**Published:** 2004-04-07

**Authors:** Michele Walters, Jeremy J Midgley, Michael J Somers

**Affiliations:** 1 Conservation Ecology Department, University of Stellenbosch, Private Bag X1, Matieland, 7602, South Africa; 2 Department of Botany, University of Cape Town, Private Bag, Rondebosch, South Africa; 3 Applied Behaviour and Ecology Lab, Department of Zoology, University of Transkei, Private Bag X1, Umtata, 5117, South Africa

## Abstract

**Background:**

While fire has been used in some instances to control the increase of woody plants, it has also been reported that fire may cause an increase in certain fire-tolerant *Acacia *tree species. This study investigated germination of *Acacia karroo*, *A. luederitzii *and *Dichrostachys cinerea*, thought to be increasing in density, as well as the historically successful encroaching woody species, *A. nilotica, *in savanna grassland, Hluhluwe-iMfolozi Park, South Africa. *A. karroo *is thought to be replacing *A. nilotica *as the dominant microphyllous species in the park. We tested the hypothesis that observed increases in certain woody plants in a savanna were related to seed germination and seedling establishment. Germination is compared among species for burnt and unburnt seeds on burnt and unburnt plots at three different locations for both hot and cool fires.

**Results:**

*Acacia karroo *showed higher germination (*A. karroo *5.1%, *A. nilotica *1.5% and *A. luederitzii *5.0%) levels and better establishment (*A. karroo *4.9%, *A. nilotica *0.4% and *A. luederitzii *0.4%). Seeds of the shrub *Dichrostachys cinerea *did not germinate in the field after fire and it is thought that some other germination cue is needed. On average, burning of *A. karroo*, *A. nilotica *and *A. luederitzii *seeds did not affect germination. There was a significant difference in the germination of burnt seeds on burnt sites (4.5%) and burnt seeds on unburnt plots (2.5%). Similarly, unburnt seeds on unburnt sites germinated better (4.9%) than unburnt seeds on burnt sites (2.8%).

**Conclusion:**

We conclude that a combination of factors may be responsible for the success of *A. karroo *and that fires may not be hot enough or may occur at the wrong time of year to control *A. karroo *establishment in HiP. Although germination and establishment of *A. karroo *was higher than for *A. nilotica *a competitive advantage after fire could not be shown.

## Background

The increasing density in the woody component of savannas has been widely reported [1-5] with special mention being made of *Acacia karroo *Hayne [6,7] and *A. nilotica *(L.) Willd. Ex Del. subsp. *kraussiana *(Benth.) Brenan, [8,9]. in some areas, as major contributors to the phenomenon. In Hluhluwe-iMfolozi Park *Dichrostachys cinerea *(L.) Wight & Arn. and *A. luederitzii *Engl. var. *retinens *(Sim) Ross & Brenan are also thought to contribute to this phenomenon.

In hard seeded legumes dormancy is broken by rupturing part of the seed coat. The rupturing of the seed coat may be induced by heat from fire [10] enabling water to enter the seed and start the process of germination. Many studies have confirmed a release of legume seeds from dormancy after fire [10-17]. Fire temperature or intensity also has an effect on the germination of seeds [17,18] and low intensity fires may not be enough to break dormancy of hard-seeded legumes [19]. In other cases lower fire temperatures are preferable for germination with an increase in fire temperature causing seed mortality [18].

While some studies report that a decrease in grass cover favours the establishment of woody seedlings due to reduced competition [20,21], others [6,22] challenge these findings. These differences may however, be a result of species reacting differently to fire or competition.

Some *Acacia *species are shade intolerant resulting in decreased seedling establishment in shady areas [20,23,24]. Other *Acacia *species have been found to be tolerant of low light conditions and may even experience increased seedling survival [6].

The frequency of fires may affect the direction of change in woody plant density [5]. While it has been suggested that fire may increase *Acacia *densities [10], it is also used to clear acacias from grassland [25]. This contradictory situation in the literature concerning the effect of fire necessitates further research, as it is clear that continuous use of incorrect burning practices may have disastrous consequences.

This study investigated the direct (heat) and indirect (grass removal) effects of fire on seed germination and seedling establishment of *A. nilotica*, *A. karroo*, *A. luederitzii *and *Dichrostachys cinerea *in Hluhluwe-iMfolozi Park (HiP), where an increase in woody plant density over the past 40 years has been reported [26-28]. It has also been reported that *A. karroo *is apparently replacing *A. nilotica *as the dominant microphyllous element [27,28].

This study reports on the effects of burning, fire intensity and burning of sites on germination; burning, fire intensity, burning of sites and grass length (shade) on seedling establishment and specific species responses to treatments (treatment species interactions).

## Results

### Germination

**Figure 2:**
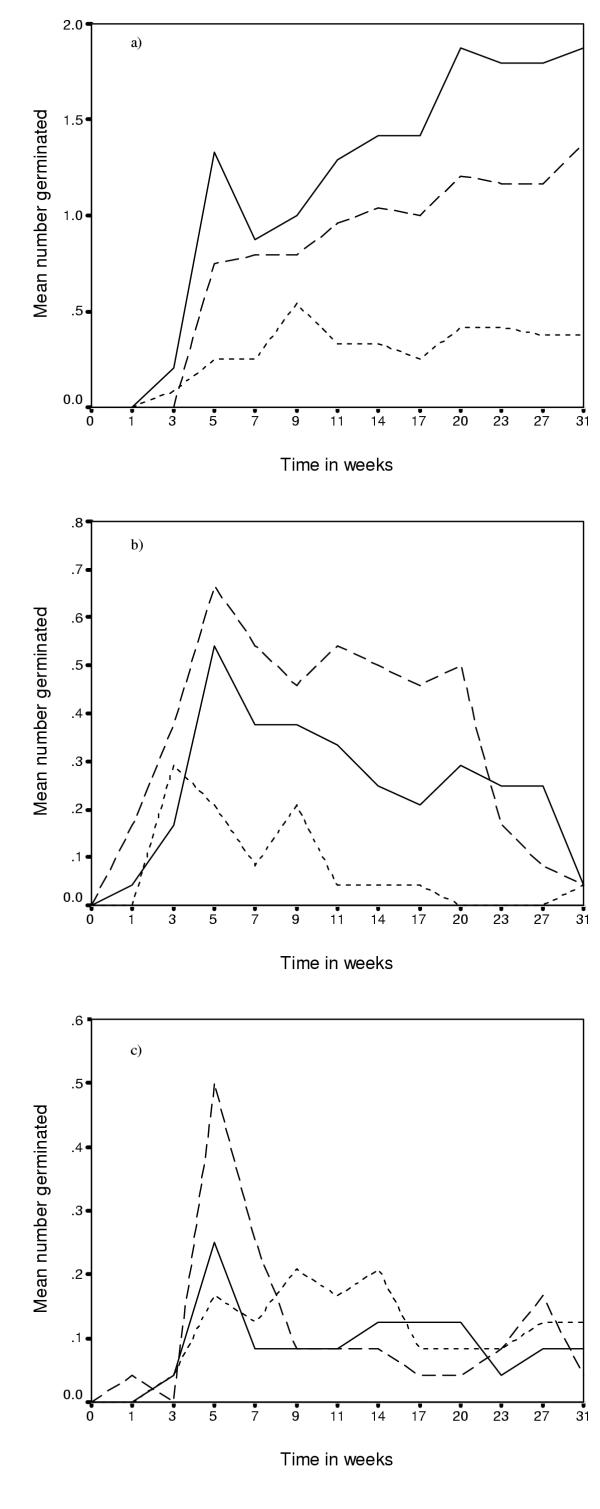
Mean number of germinated seeds recorded over a 31-week period at three different locations in HiP for a) *Acacia karroo*, b) *Acacia luederitzii *and c) *Acacia nilotica*.

None of the seeds of *D. cinerea *germinated in the field and it was therefore excluded from the model for the field experiment. Testing for differences among treatments was based on the maximum number of seedlings for each species at each location over the 31-week period (Figure 2). A description of the factors used in both the germination and establishment models is given in Table 1.

**Table 1:** Descriptions of factors used in the models and number of seeds used for each factor.

	Germination				Establishment			
Factor/Description	Total number of seeds	Number not germinated	Number germinated	Percent germinated	Total number of seeds	Number not established	Number established	Percent established

Total	4073	3923	150	3.68	4062	3966	96	2.36
Location								
Seme	1348	1287	61	4.53	1337	1302	35	2.62
Nombali	1364	1300	64	4.69	1364	1316	48	3.52
Le Dube	1361	1336	25	1.84	1361	1348	13	0.96
Species								
*A. karroo*	1786	1695	91	5.10	1788	1701	87	4.87
*A. luederitzii*	720	684	36	5.00	707	704	3	0.42
*A. nilotica*	1567	1544	23	1.47	1567	1561	6	0.38
Burnt or unburnt								
burnt	2021	1950	71	3.51	2030	1985	45	2.22
unburnt	2052	1973	79	3.85	2032	1981	51	2.51
Tall or short grass								
tall (>0.1 m)	2039	1961	78	3.83	2041	1993	48	2.35
short	2034	1962	72	3.54	2021	1973	48	2.38
Site burnt or unburnt								
burnt	2052	1977	75	3.65	2052	2003	49	2.39
unburnt	2021	1946	75	3.71	2010	1963	47	2.34

The ratio of the model deviance to the degrees of freedom was small (0.29) indicating that the model was a good fit. Location and species were the only main effects significantly affecting germination (Table 2). *Acacia karroo *had the highest germination of all species (Table 1).

**Table 2:** Statistics indicating significance of the factors and interactions on germination. Significant factors are in bold.

Factor	df	Log-likelihood	Chi-Square	Wald Stat.	P
**Location**	**2**	**-587.555**	**13.915**	**11.547**	**0.003**
**Species**	**2**	**-597.790**	**34.386**	**25.394**	**0.000**
Burnt status	1	-582.073	2.951	2.822	0.093
Grass length	1	-580.622	0.050	0.050	0.822
Site burn status	1	-580.608	0.021	0.021	0.885
Location*species	4	-582.584	3.974	3.827	0.430
Location*burn status	2	-582.929	4.664	4.373	0.112
Location*grass length	**2**	**-586.296**	**11.397**	**10.812**	**0.004**
Location*site burn status	2	-580.703	0.212	0.211	0.900
Species*burn status	2	-581.173	1.151	1.145	0.564
Species*grass length	2	-581.019	0.843	0.837	0.658
Species*site burn status	2	-583.309	5.424	5.166	0.076
Burn status*grass length	1	-580.767	0.340	0.341	0.559
Burn status*site burn status	**1**	**-585.060**	**8.926**	**8.656**	**0.003**
Grass length*site burn status	**1**	**-587.530**	**13.866**	**13.082**	**0.000**

Interaction terms that had a significant effect on germination were, location × grass length, burn status × site burn status and grass length × site burn status (Table 2). Germination of burnt seeds in burnt sites (4.5%) was significantly higher than that of burnt seeds in unburnt sites (2.5%). Similarly, unburnt seeds in unburnt sites had a higher germination percentage (4.9%) than unburnt seeds in burnt sites (2.8%).

The estimated odds of germination and their associated probabilities for the factors and their interactions are given in Additional file 1. The odds ratios for significant effects were calculated. Thus a comparison between *A. karroo *and *A. nilotica *with regards to seeds germinating was made, where



Thus the odds of germinating are four times more for *A. karroo *than for *A. nilotica*. Similarly *A. nilotica *was four times less likely to germinate than *A. luederitzii *while *A. karroo *and *A. luederitzii *had the same odds of germinating. Differences in germination among species for the various treatments are given in Table 3.

**Table 3:** A comparison of germination among species for the different levels of the main factors.

		*A. karroo*				*A. luederitzii*				*A. nilotica*			
Factor/description	n	Total count	Not germ	germ	% erm	Total count	Not germ	germ	%germ	Total count	Not germ	germ	% germ

Location*Species													
Seme	48	591	558	33	5.91	240	224	16	7.14	517	505	12	2.38
Nombali	48	596	551	45	8.17	240	227	13	5.73	528	522	6	1.15
Le Dube	48	599	586	13	2.22	240	233	7	3	522	517	5	0.97
Burnt or unburnt*Species													
burnt	72	886	839	47	5.6	360	344	16	4.65	775	767	8	1.04
unburnt	72	900	856	44	5.14	360	340	20	5.88	792	777	15	1.93
Tall or short grass*Species													
tall	72	895	851	44	5.17	360	340	20	5.88	784	770	14	1.82
short	72	891	844	47	5.57	360	344	16	4.65	783	774	9	1.16
Site burnt or unburnt*Species													
yes	72	900	854	46	5.39	360	338	22	6.51	792	785	7	0.89
no	72	886	841	45	5.35	360	346	14	4.05	775	759	16	2.11

There was 2.3 times less germination at Le Dube than at Nombali and 2.6 times less at Le Dube than at Seme. Germinations were 1.2 times more likely at Seme than at Nombali.

### Seedling establishment

**Figure 3:**
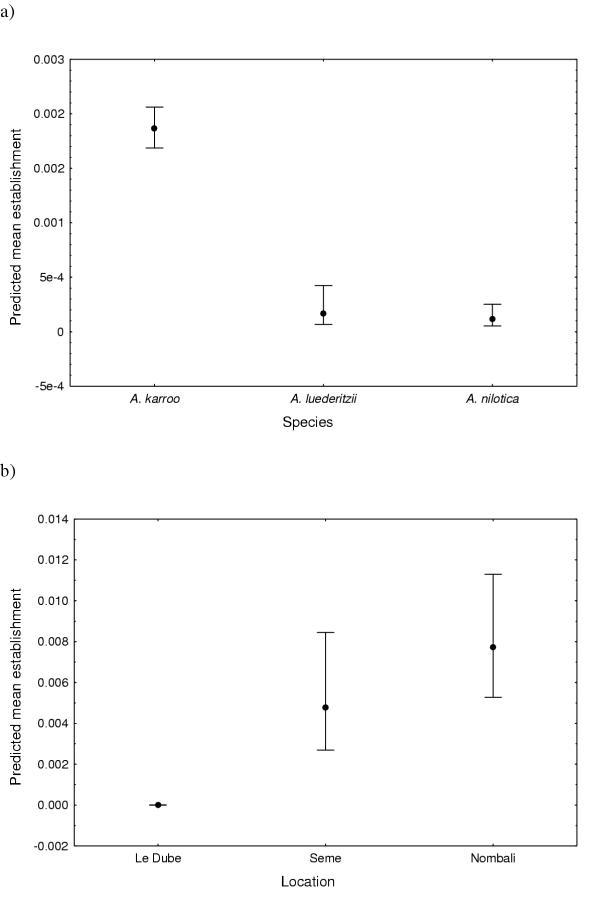
Predicted mean establishment for the significant main effects of a) species and b) location. Vertical error bars show 95% confidence limits.

The ratio of the model deviance to the degrees of freedom was small (0.17) indicating that the model fitted the data well. Location and species were the only main effects significantly affecting establishment in the field (Table 4 & Figure 3). *Acacia karroo *showed significantly higher percentage establishment than any of the other species (Additional file 2, Table 5 & Figure 3).

**Table 4:** Statistics indicating significance of factors and interactions on establishment. Significant factors are indicated in bold.

Factor	df	Log-likelihood	Chi-Square	p
**Location**	**2**	**-443.238**	**22.292**	**<0.001**
**Species**	**2**	**-395.199**	**96.079**	**<0.001**
Burnt status	1	-395.050	0.297	0.586
Grass length	1	-395.049	0.002	0.962
Site burn status	1	-395.040	0.018	0.894
Location*species	4	-391.756	6.568	0.161
Location*burn status	**1**	**-380.850**	**21.812**	**<0.001**
Location*grass length	**2**	**-373.542**	**14.617**	**<0.001**
Location*site burn status	**2**	**-367.865**	**11.353**	**0.003**
Species*burn status	2	-367.468	0.795	0.672
Species*grass length	2	-367.344	0.248	0.884
Species*site burn status	2	-367.180	0.329	0.848
Burn status*grass length	1	-366.723	0.913	0.339
Burn status*site burn status	**1**	**-360.267**	**12.913**	**<0.001**
Grass length*site burn status	**1**	**-351.784**	**16.965**	**<0.001**

**Table 5:** A comparison of establishment among species for the different levels of the main factors

		*A. karroo*				*A. luederitzii*				*A. nilotica*			
Factor/Description	n	Total count	Not estab	estab	% estab	Total count	Not estab	estab	% estab	Total count	Not estab	estab	%estab

Location*Species													
Le Dube	48	599	590	9	1.53	240	239	1	0.42	522	519	3	0.58
Nombali	48	598	553	45	8.14	238	237	1	0.42	528	526	2	0.38
Seme	48	591	558	33	5.91	229	228	1	0.44	517	516	1	0.19
Burnt or													
unburnt*Species													
Burnt	72	886	843	43	5.1	347	346	1	0.29	797	796	1	0.13
Unburnt	72	902	858	44	5.13	360	358	2	0.56	770	765	5	0.65
Tall or short													
grass*Species													
Tall	72	897	854	43	5.04	360	358	2	0.56	784	781	3	0.38
Short	72	891	847	44	5.19	347	346	1	0.29	783	780	3	0.38
Site burnt or													
unburnt*Species													
Yes	72	900	855	45	5.26	360	359	1	0.28	792	789	3	0.38
No	72	888	846	42	4.96	347	345	2	0.58	775	772	3	0.39

**Figure 4:**
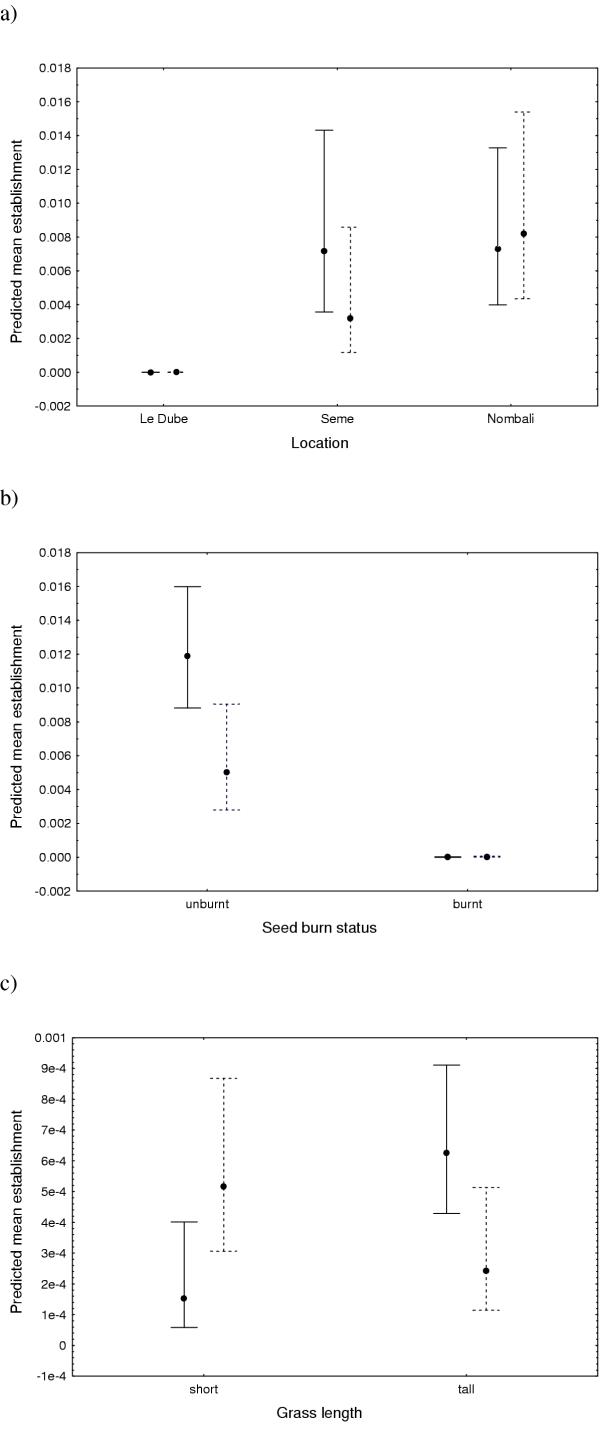
Predicted mean establishment for significant interactions of site burn status and a) location, b) seed burn status and c) grass length. The solid line represents unburnt sites and the dotted line burnt sites. Vertical error bars show 95% confidence limits.

Interaction terms, location × burn status, location × grass length, location × site burn status, burn status × site burn status and grass length × site burn status had a significant effect on establishment (Table 4) (Figure 4).

Additional file 2 gives the estimated odds of non-establishment and their associated probabilities for the factors and their interactions. The odds ratios for significant effects were calculated and are given (see Additional file 3).

*Acacia karroo *was 16.2 times more likely to establish than *A. nilotica. *Similarly *A. luederitzii *was 1.4 times more likely to establish than *A. nilotica *while *A. karroo *had 11.2 times more chance of establishing than *A. luederitzii. *Species differences in establishment for the various treatments are given in Table 5.

The odds of establishment were 8046.2 times less at Le Dube than at Nombali and 5850.5 times less at Le Dube than at Seme. 1.4 times more seedlings were likely to establish at Nombali than at Seme.

## Discussion

The lack of germination of *D. cinerea *in the field suggests that some disturbance other than fire is needed to cause a release from dormancy and commence germination.

Germination of all species in the field was low. As the seeds relied on cotyledons for food, soil moisture may have been a limiting factor. As rainfall was not recorded, this should be kept in mind when interpreting the results. Five point one percent of *A. karroo *seeds germinated, which was higher than the other two species. Story [29] found similar levels of germination for *A. karroo, *with 6.6% of seeds germinating under natural conditions in the field. He also found that *A. karroo *germination was erratic, with germinations still being recorded after 423 days. This was similar to what was found in this study, with the number of *A. karroo *seedlings still increasing until the end of the experiment. *Acacia nilotica *also showed dormancy with sporadic germination events over the 31-week period. *Acacia luederitzii *did not show dormancy with most germinations taking place in the first 3 weeks of the experiment. *Acacia nilotica *has a thick seed coat, which could account for it's poor level of germination. One would predict increased germination of burnt seeds due to a breaking of dormancy [18], but this was not the case. A possible explanation is that the temperature of the fires in this study, though not measured, might not have been sufficient to break dormancy in this species. Some *Acacia *species are temperature specific, suggesting a temperature threshold for germination [18,20]. This is unlikely in this case as Radford *et al*. [30] found *A. nilotica *seeds to be highly vulnerable to fire with a 80% mortality of seeds on the soil surface. The current study, however, found no difference in germination between burnt and unburnt seed or seeds burnt at different temperatures. This finding is inconsistent with the recent study by Kanz [20] who found increased seed germination in low fires compared to the control as well as that of Okello and Young [31] who found increased germination of unburnt seeds. Auld & O'Connell [18] had similar results to that of Kanz [20] with strong germination responses to heat.

Location had a significant effect on germination with Le Dube having very low germination overall and Seme having the most germinations. Germination at Nombali and Seme were similar. Site-specific effects may be attributed to various factors such as microclimate or soil type. Sites may also have different water infiltration rates and runoff, which may result in differences in germination levels. Okello and Young [31], however, found that soil type did not affect germination or establishment of *Acacia drepanolobium *in Kenya.

The current study did not find a difference in the number of seedlings in burnt and unburnt patches. While neither burning of seeds nor burning of sites had any effect on germination, the interaction factor proved significant with unburnt seeds showing increased germination in unburnt sites as did burnt seeds in burnt sites. Kanz [20] also found greater seedling emergence of unburnt seeds in unburnt areas. This might be a result of burnt seeds imbibing faster than unburnt seeds, possibly making them more susceptible to rot. Burnt seeds would therefore show poorer germination in unburnt areas due to increased moisture retention. Similarly, unburnt seeds would require more moisture to imbibe, resulting in decreased germination in burnt areas due to decreased moisture in these open areas.

Whilst more seeds germinated in short grass at both Le Dube and Nombali, those at the short-grass site (Seme) had higher levels of germination in tall grass sites. The short grass site at Seme is a white rhinoceros (*Ceratotherium simum*) grazing lawn with very short grass, which may lead to seeds losing moisture through more direct sunlight. This suggests a similar pattern to the seed burn × site burn interaction. The tall grass site at Seme had higher germination than any of the other tall or short grass sites. This may be due to possible site-specific effects mentioned earlier.

There was also an interaction between grass length and site burn with seeds in burnt, short grass showing higher germination than those in burnt, tall grass and unburnt sites showing higher germination in tall grass. As half of the seeds on a burnt or unburnt site were burnt themselves, it is possible that this interaction is due to temperature sensitivity in seeds. Burning in tall grass (hotter fires) may be detrimental to the germination of seeds [18] while cooler fires may be sufficient to break dormancy and cause germination. Higher germinations in unburnt tall grass areas suggest a shade effect. This is not certain, as the effects of shade and grass competition were not separated in this study. *Acacia karroo *has however been reported as having an increased ability to survive in shade with recruitment of seedlings being dependent on moisture availability [6]. Tall grass species may retain more moisture than short grass species, affording seeds a better opportunity for germination.

No species factor interactions were observed suggesting that though species had different germination levels, they did not respond differently to the treatments.

The same factors and interactions found to be significant influences on germination were found to influence establishment. This was expected as increased germination for these treatments would result in better establishment. The interaction patterns for most of the treatments, however, were different to those of the germination model. Owing to the low levels of germination, interspecific and intraspecific competition was thought to play a minor role in seedling establishment.

Le Dube again had the least seedlings at 31 weeks while Nombali had the best establishment. Seme, which had the highest level of germination, had establishment levels somewhere between that of the other two sites. It is again suggested that this may be due to soil or rainfall factors. Forty-five out of forty-eight seedlings established at Nombali and thirty-three out of thirty-five at Seme were *A. karroo *seedlings. This species is known to be dependent on moisture availability for survival [6] and these two sites might have better water retaining ability than Le Dube.

At week 31, 87 *A. karroo *seedlings had established as opposed to six of *A. nilotica *and three of *A. luederitzii. *The high germination, but poor survival of *A. luederitzii *suggests that the absence of this species in the Hluhluwe section of HiP is not due to seed limitation or germinability, but possibly due to environmental factors decreasing its ability to establish. The differences in seedling survival between species are consistent with those reported by Kanz [20] who found higher seedling survival for *A. karroo *than *A. nilotica*.

The location × grass length interaction revealed the same patterns as for germination with regards to Nombali and Seme with Seme showing better establishment in tall grass and Nombali showing better establishment in short grass. There was no difference between establishment on tall and short grass at Le Dube. The short grass site at Nombali had the highest number of seedlings surviving at week 31.

The grass length × site burn interaction displayed the same patterns as for the germination model, but this was not the case for the seed burn status × site burn status interaction. While unburnt seeds still did well on unburnt sites, burnt and unburnt seeds showed decreased establishment on burnt sites suggesting that, as a result of increased irradiance, burnt (open) sites may not hold sufficient moisture for seedlings to survive.

The interaction effects found to be significant for establishment only, both suggest the importance of fire temperature. Location × seed burn status and location × site burn status could both relate to the different grass lengths, and thus specific fire temperatures, at the three sites. Temperature sensitivity in *Acacia *species have been reported elsewhere [11,14,17,20]. Kanz [20] found increased survival and growth in burnt areas. In this study, Nombali was the only location to have higher establishment on burnt sites, while Seme had increased establishment on unburnt sites and Le Dube very little establishment overall. In general, however, this study found no difference in establishment in burnt and unburnt areas.

Chirara, Frost & Gwarazimba [7] found that intensity of grass defoliation does not affect seedling establishment of *A. karroo *during the first year. Similarly, there was no difference in establishment of *A. karroo *in burnt or unburnt and tall or short grass sites. Smith & Goodman [32] reported that *A. nilotica *seedlings, however, almost exclusively occurred away from canopy cover, suggesting an inability to establish in shaded environments. *Acacia tortilis *also showed a greater proportion of established seedlings in open than shaded areas [23]. We did not find a difference in establishment of *A. nilotica *in tall and short grass, but its establishment was so low that no real prediction can be made.

## Conclusions

Seedling establishment of *A. karroo *is strongly moisture dependent [6] and one would expect that *A. karroo *is more likely to invade moist rather than semi-arid grassland. This suggests that Hluhluwe Game Reserve, being an area with moist grassland, would be more prone to invasion by *A. karroo*. It has also been reported that *A. karroo *has the ability to withstand fire [17]. A combination of these factors may contribute to the success of *A. karroo *in the field and may be the reason for *A. karroo*'s success over *A. nilotica *as the most important encroaching *Acacia *species in HiP at present. The literature does, however, suggest that high intensity fires may result in seed mortality [18,20]. It has, however, been reported that *A. karroo *seedlings survive fires from as little as 12 months of age [29]. Therefore, if fires are not hot enough to kill the seeds allowing them to germinate and seedlings to establish, management burns in the following year may not be useful in its attempt to control the establishment of this species. Back fires have higher fire intensities than head fires [20]. We therefore suggest that backfires be used during management burns and that fire frequency be increased in suitable areas in an attempt to slow down the rate of encroachment by *A. karroo. *It has been reported that spring burns are the most effective ([33] in [29]) and this should be taken into account.

## Methods

### Study site

The study was done in HiP, KwaZulu-Natal, South Africa (28°00' – 28°26' S, 31°43' – 32°09'E). HiP is a 960 km^2 ^fenced protected area comprising the former Hluhluwe and iMfolozi Game Reserves, and the corridor of land that links the areas. The park has a moderate coastal climate, ranges in altitude from 60 – 750 m above sea level [34] and has a summer rainfall ranging between 760 and 1250 mm per annum. Hluhluwe Game Reserve has a mean annual rainfall of 990 mm, while iMfolozi Game Reserve has a mean annual rainfall of 720 mm [34]. Periodic fluctuations in above or below average annual rainfall occur, resulting in wet and dry spells of approximately nine years [35]. The range in average monthly temperature is between 13 and 33°C [36].

Most of Hluhluwe Game Reserve is found on rocks of the Ecca and Beaufort series with some basalt in the east [37]. King [37] identified seven geological formations: (1) the Granite-Gneiss base, (2) the Table Mountain sandstone, (3) the Dwyka tillite, (4) The Ecca and Beaufort series, (5) the Stormberg series, (6) fault breccias and (7) recent deposits.

The main soils types associated with the Ecca and Beaufort series are Swartland and Sterkspruit, while areas of Shortlands, Milkwood and Bonheim series are found in association with the dolerite regions [34]. They also report that shallow Mispah soils occur extensively in the reserve.

The vegetation in the park has been described as bushveld – savannah comprising five broad vegetation types [38]. The thickets are wooded groups of similar-sized, small (usually less than three metres) trees of mainly one species that grows densely to the exclusion of other species. The thornveld consists of scattered thorn trees on grassland with deciduous, broad-leaved trees standing out above the thorn trees while the woodlands are densely wooded areas of tall trees that may contain many different, mainly broadleaved species. The well drained, shallow soils of the rocky outcrops support scattered trees of various sizes, while the termite mounds are nutrient rich patches sustaining dense clumps of trees that form small, wooded islands [38]. Locally the reserve is described as Natal Lowveld Bushveld and falls within the savanna biome [39].

The field experiment took place in the Hluhluwe and Corridor sections of the HiP. *Acacia luederitzii *occurs in large numbers in certain areas of the iMfolozi part of the reserve but is mostly absent from the Hluhluwe and Corridor sections. *Acacia nilotica, A. karroo *and *D. cinerea *are found throughout the park. As opposed to the scattered trees found in iMfolozi, *A. nilotica *covers extensive areas of Hluhluwe and the Corridor and is usually found below the 300 m contour [34]. Whateley & Porter [34] described an *A. karroo – D. cinerea *induced thicket throughout the area, but particularly in the Corridor and Hluhluwe Reserves. *Acacia luederitzii *seeds used in this study were therefore collected in iMfolozi Game Reserve while those of the other species were collected in Hluhluwe.

### Germination

**Figure 1:**
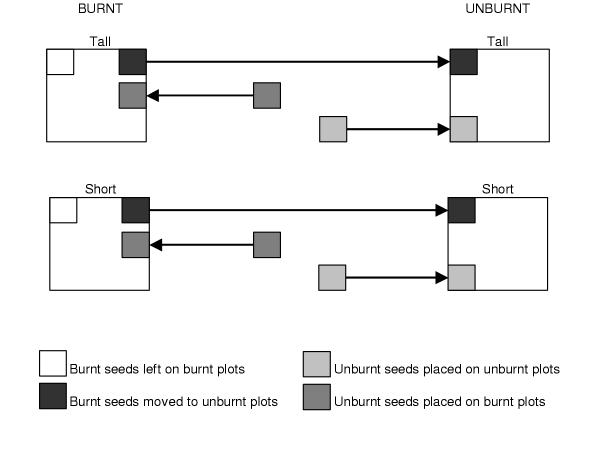
Diagrammatical representation of the experimental design used to test the effect of fire on seed germination and establishment. Arrows indicate movement of seeds between burnt/unburnt tall/short grass plots.

The effect of fire, fire intensity and burning of sites on the germination of seeds of *A. nilotica*, *A. karroo*, *A. leuderitzii *and *D. cinerea *was tested in a field experiment. Seeds of all species were collected between May and August 2000. Parasitized seeds were extracted. Prior to planned management burns, six groups of seeds were placed in tall grass (taller than 0.10 m) and six in short grass (shorter than 0.10 m) at three locations (Nombali, Seme and Le Dube). Tall grass produces hotter fires than short grass due to increased fuel load, which increases available heat energy [40]. Sites were cleared of existing pods/ seeds prior to the experiment and as podding season was over, no uncontrolled additions are expected to have occurred. *Dichrostachys cinerea *seeds were only put out at Seme and Nombali. Each group contained 22 *A. nilotica*, 25 *A. karroo*, 10 *A. leuderitzii *and 10 *D. cinerea *seeds. Seeds were placed on the soil surface a day before each of the burns (Nombali two days before). This is considered the natural situation for the seeds with soil stored seed banks being virtually non-existent [41]. Seme and Le Dube were burnt on 2 October and Nombali on 30 September 2000 shortly before the start of spring rains and natural seed release. After the burns, three of the groups of burnt seeds were removed from the tall and short grass and placed on unburnt tall and short grass sites at the same location respectively. Three groups of unburnt seeds were then added to each of the tall and short grass sites. A 13 mm mesh cage with 18 cm × 18 cm × 18 cm sides was used to protect each group of seeds and any germinated seedlings from rodent and herbivore predation. Cages were placed at half metre intervals and seeds placed on the soil surface in a group in the middle of each cage Seeds were considered to be germinating when a root started showing. A diagrammatical representation of the experiment is given in Figure 1. Germination was recorded at 1, 3, 5, 7, 9, 11, 14, 17, 20, 23, 27 and 31 weeks. The experiment ended in May 2001.

We thus applied 96 possible seed treatment combinations for investigating factors affecting germination in the field (4 species × 2 burn treatments × 3 locations × 2 location burn treatments × 2 fire intensities).

### Seedling establishment

To test the effect of fire, fire intensity, burning of sites and grass length (shade) on seedling establishment of *A. nilotica*, *A. karroo*, *A. leuderitzii *and *D. cinerea*, data as on week 31 of the field experiment described above were used. Seedlings were considered to be established when they were rooted in the ground and the cotyledons replaced with leaves. Establishment was based on the total number of seeds.

### Data analysis

The "STATISTICA^®^" [42] Generalized Linear Model (GLZ) module was used to construct linear logistic models for germination and establishment proportions as response variables for the field experiment. As data were recorded as presence (1) or absence (0) of seedlings, a binomial distribution was assumed [43]. In both cases, main effects and second order interactions were included in the model. The logit model may therefore be written as follows:



where

 = the log of variable 1 and 2 at different levels of the factors as given below

λ' = the overall mean effect of the categories

 = the effect of the *j*th species (*j *= *A. karroo*, *A. luederitzii*, *A. nilotica*, *D. cinerea*)

 = the effect of the *k*th location (*k *= Le Dube, Nombali, Seme)

 = the effect of the *l*th seed burn status (*l *= burnt, unburnt)

 = the effect of the *m*th grass length (*m *= short, tall)

 = the effect of the *n*th site burn status (*n *= burnt, unburnt)

 = the interaction effect between the *j*th species and the *k*th location

 = the interaction effect between the *m*th grass length and the *n*th site burn status.

The logit model may be written as a generalized linear model as follows:



where , , , , , , , , ,  and  are parameters to be estimated from the data and B, C, D, E and F refer to the explanatory variables species, location, burn status, grass length and site burnt status respectively. The estimated parameters for the GLZ were used to obtain the estimated parameters for the logit model. The estimated parameters of the odds were calculated for each factor or combination of factors (including the intercept) as the exponent of the estimated parameters of the logit model. The estimated odds of germination under any condition were then calculated as the product of the estimated parameter of the odds of the intercept (estimated geometric mean odds) and the factor or combination of factors in question. The odds of germination for significant treatment combinations were compared.

The predicted number of seeds germinating and seedlings establishing as calculated with the model based on presence/absence data, were seen as being appropriate for interpretation as summaries of the data. Thus, differences in the predicted mean number of seeds germinating and seedlings establishing (given as a fraction of the total number of seeds) were illustrated graphically for each significant treatment combination.

## Authors' contributions

MW designed the experiment, participated in fieldwork, performed the statistical analysis and drafted the document. MJS participated in fieldwork, the coordination of the study and drafting of the document. JJM supervised the work and assisted in the drafting of the document. All authors read and approved the final manuscript.

## Supplementary Material

Additional File 1 The parameters of the logit model and odds, estimated odds of germination and the ratio of germination to non-germination for the factors included in the model for germination of certain *Acacia *seeds in HiP. Gives parameters of the logit model and estimated odds of germination for the various levels of the factors used. Click here for file

Additional File 2 The parameters of the logit model and odds, estimated odds of establishment and the ratio of establishment to non-establishment for the factors included in the model for establishment of certain *Acacia *species in HiP. Gives parameters of the logit model and estimated odds of establishment for the various levels of the factors used. Click here for file

Additional File 3 Odds ratios for all significant interactions of the establishment model. Compares the odds of establishment between different levels of the factors used. Click here for file
